# Supplementary material for: Detecting monogenic obesity: a systematic exome-wide workup of over 500 individuals
Source: Int J Obes (Lond). 2025 Jun 16;49(7):1400–11. doi: 10.1038/s41366-025-01819-0 (PMC12283394; doi:10.1038/s41366-025-01819-0)
Supplement: Supplementary file 1 — Supp. Fig. 1-2 and Table 2-4 [file 41366_2025_1819_MOESM1_ESM.pdf]

<u>Supplementary Information</u>

- **Supp. Fig. 1:** Flowchart describing the study's cohort selection process: *p.2*
- **Supp. Fig. 2:** Yields in further subgroups *p.3*
- **Supp. Table 1:** List of all genes included in the TruSight One Sequencing Panel (May 2014): *additional excel file*
- **Supp. Table 2:** Characteristics of patients in this study: *p.4*
- **Supp. Table 3:** Diagnostic yield in subgroups of patients: *p.5*
- **Supp. Table 4:** Comparison of affected genes in solved cases with obesity panel genes: *p.6*

Supplementary Figure 1 - Inclusion criteria

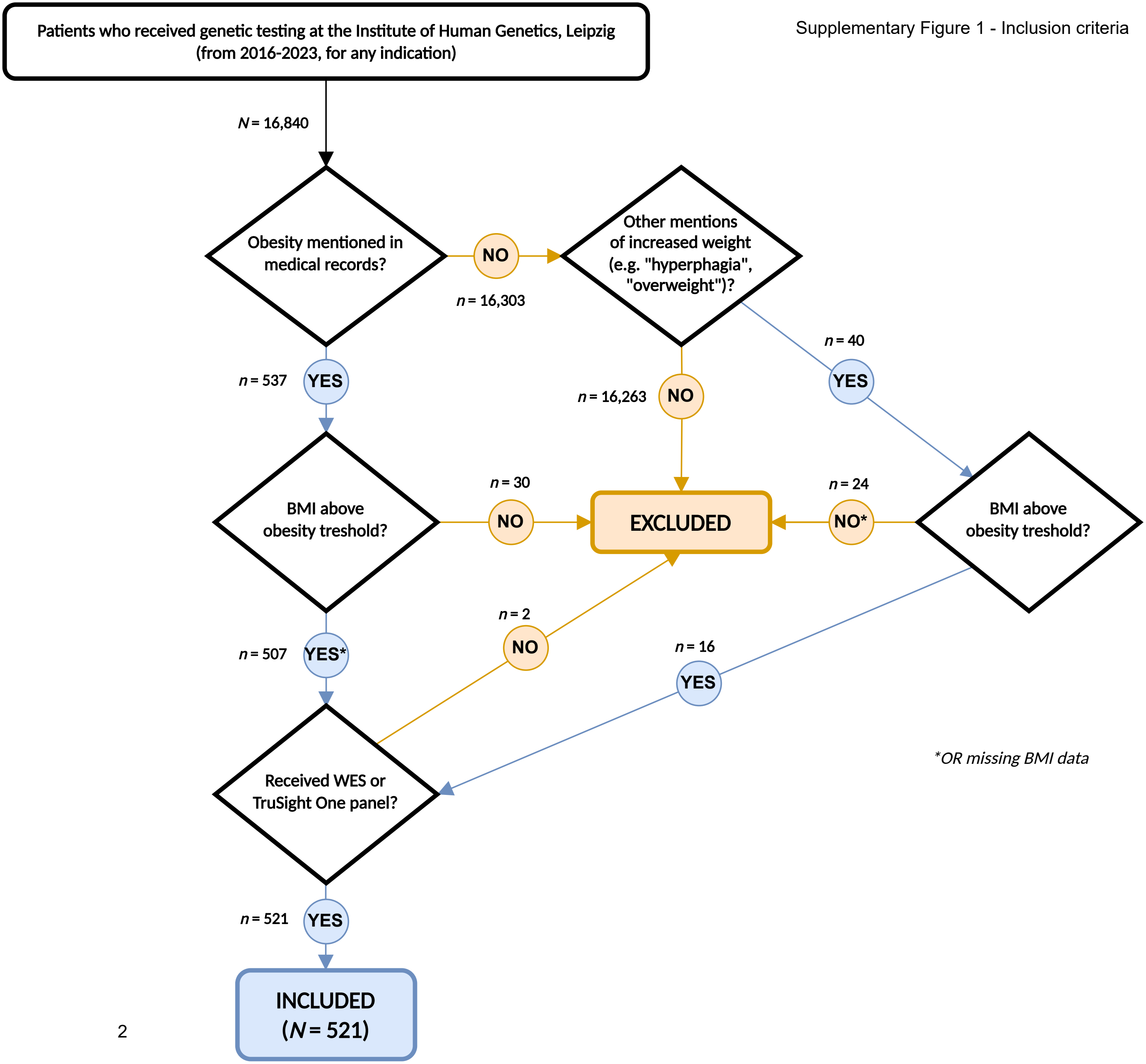

Supplementary Figure 2

A: Subgroup yields in children and adults

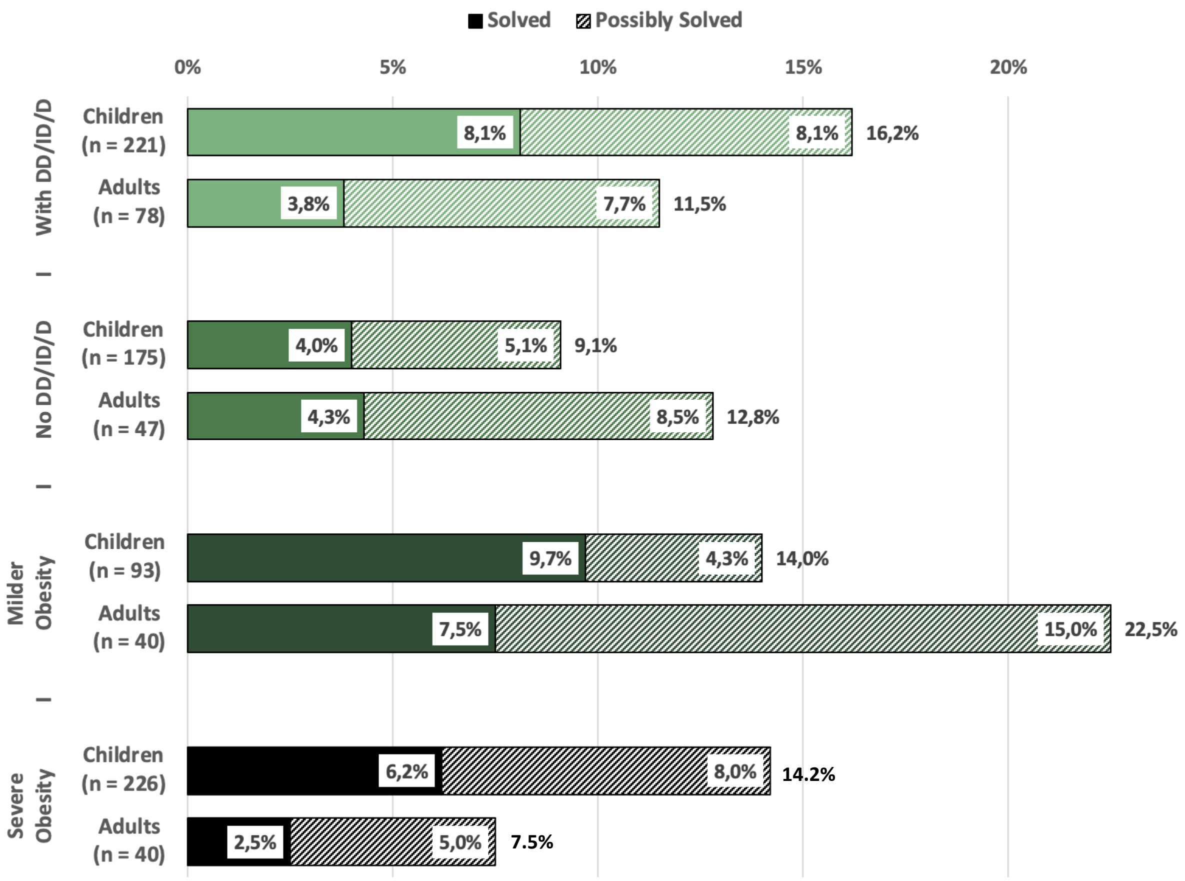

B: Yields in patients with non-syndromic obesity, depending on the severity of obesity

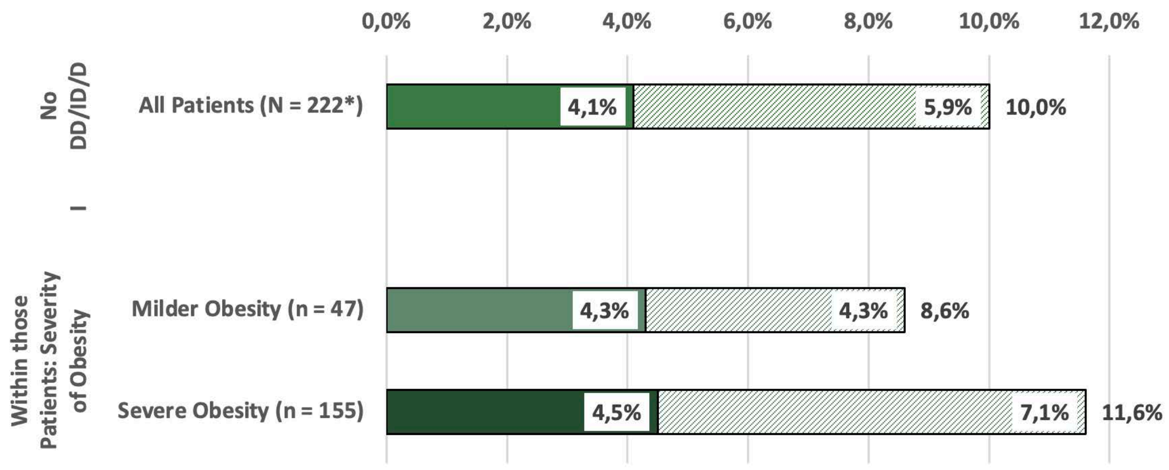

**20 patients with missing BMI data, all unsolved*

Supplementary Table 2 - Patient Characteristics

	All Patients (N = 521)		Solved (n = 30, 5.8%)		Possibly solved (n = 37, 7.1%)		Unsolved (n = 454, 87.1%)	
**Sex (n, %)**								
Male	286	54.9%	17	56.7%	23	62.2%	246	54.2%
Female	235	45.1%	13	43.3%	14	37.8%	208	45.8%
**Age (n, %)**								
Children	396	76.0%	25	83.3%	27	73.0%	344	75.8%
Adults	125	24.0%	5	16.7%	10	27.0%	110	24.2%
Mean, Median (years)	16.3, 12.8		13.8, 13.1		17.3, 14.4		16.4, 12.7	
Range (years)	0.3 – 79.8		0.4 – 50.2		1.6 – 57.6		0.3 – 79.8	
**Other Symptoms (n, %)**								
Additional DD/ID/D	299	57.4%	21	70.0%	24	64.9%	254	55.9%
No DD/ID/D	222	42.6%	9	30.0%	13	35.1%	200	44.1%
**Severity of Obesity (n, %)**								
Milder Obesity	133	25.5%	12	40.0%	10	27.0%	111	24.4%
Severe Obesity	266	51.1%	14	46.7%	20	54.1%	232	51.1%
BMI unknown	122	23.4%	3	10.0%	7	18.9%	112	24.7%

	Children (n = 396)		Solved (n = 25)		Possibly solved (n = 27)		Unsolved (n = 344)	
**Sex (n, %)**								
Male	227	57.3%	15	60.0%	15	59.3%	197	57.3%
Female	169	42.7%	10	40.0%	12	44.4%	147	42.7%
**Age (years)**								
Mean, Median	10.1, 10.2		10.5, 11.4		9.8, 8.8		10.1, 10.3	
Range	0.3 – 17.9		0.4 – 17.8		1.6 – 17.3		0.3 – 17.9	
**Other Symptoms (n, %)**								
Additional DD/ID/D	221	55.8%	18	72.0%	18	66.7%	185	53.8%
No DD/ID/D	175	44.2%	7	28.0%	9	33.3%	159	46.2%
**Severity of Obesity (n, %)**								
Milder Obesity	93	23.5%	9	36.0%	4	14.8%	80	23.3%
Severe Obesity	226	57.1%	14	56.0%	18	66.7%	194	56.4%
BMI unknown	77	19.4%	2	8.0%	5	18.5%	70	20.3%

	Adults (n = 125)		Solved (n = 5)		Possibly solved (n = 10)		Unsolved (n = 110)	
**Sex (n, %)**								
Male	59	47.2%	2	40.0%	8	80.0%	49	44.5%
Female	66	52.8%	3	60.0%	2	20.0%	61	55.5%
**Age (years)**								
Mean, Median	36.2, 34.1		31.0, 26.1		37.5, 36.9		36.3, 34.0	
Range	18 – 79.8		18.1 – 50.2		23.7 – 57.6		18 – 79.8	
**Other Symptoms (n, %)**								
Additional DD/ID/D	78	62.4%	3	60.0%	6	60.0%	69	62.7%
No DD/ID/D	47	37.6%	2	40.0%	4	40.0%	41	37.3%
**Severity of Obesity (n, %)**								
Milder Obesity	40	32.0%	3	60.0%	6	60.0%	31	28.2%
Severe Obesity	40	32.0%	1	20.0%	2	20.0%	37	33.6%
BMI unknown	45	36.0%	1	20.0%	2	20.0%	42	38.2%

Supplementary Table 3 - Yield per Subgroup

	All Patients (N = 521)		Solved (n = 30)		Permutation Test (p-value)	Possibly solved (n = 37)		Unsolved (n = 454)	
**All Patients (N, %)**	521	100%	30	5.8%		37	7.1%	454	87.1%
**Sex (n, %)**									
Male	286	54.9%	17	5.9%	0.499	23	8.0%	246	86.0%
Female	235	45.1%	13	5.5%		14	6.0%	208	88.5%
**Age (n, %)**									
Children	396	76.0%	25	6.3%	0.231	27	6.8%	344	86.9%
Adults	125	24.0%	5	4.0%		10	8.0%	110	88.0%
Mean, Median (years)	16.3, 12.8		13.8, 13.1			17.3, 14.4		16.4, 12.7	
Range (years)	0.3 – 79.8		0.4 – 50.2			1.6 – 57.6		0.3 – 79.8	
**Other Symptoms (n, %)**									
Additional DD/ID/D	299	57.4%	21	7.0%	0.105	24	8.0%	254	84.9%
No DD/ID/D	222	42.6%	9	4.1%		13	5.9%	200	90.1%
**Severity of Obesity (n, %)**									
Milder Obesity	133	25.5%	12	9.0%	0.147	10	7.5%	111	83.5%
Severe Obesity	266	51.1%	15	5.6%		20	7.5%	231	86.8%
BMI unknown	122	23.4%	3	2.5%		7	5.7%	112	91.8%

	Children (n = 396)		Solved (n = 25)		Permutation Test (p-value)	Possibly solved (n = 27)		Unsolved (n = 344)	
**All Children (n, %)**	396	100.0%	25	6.3%		27	6.8%	344	86.9%
**Sex (n, %)**									
Male	227	57.3%	15	6.6%	0.479	15	6.6%	197	86.8%
Female	169	42.7%	10	5.9%		12	7.1%	147	87.0%
**Age (years)**									
Mean, Median	10.1, 10.2		10.5, 11.4			9.8, 8.8		10.1, 10.3	
Range	0.3 – 17.9		0.4 – 17.8			1.6 – 17.3		0.3 – 17.9	
**Other Symptoms (n, %)**									
Additional DD/ID/D	221	55.8%	18	8.1%	0.068	18	8.1%	185	83.7%
No DD/ID/D	175	44.2%	7	4.0%		9	5.1%	159	90.9%
**Severity of Obesity (n, %)**									
Milder Obesity	93	23.5%	9	9.7%	0.195	4	4.3%	80	86.0%
Severe Obesity	226	57.1%	14	6.2%		18	8.0%	194	85.8%
BMI unknown	77	19.4%	2	2.6%		5	6.5%	70	90.9%

	Adults (n = 125)		Solved (n = 5)		Permutation Test (p-value)	Possibly solved (n = 10)		Unsolved (n = 110)	
**All Adults (n, %)**	125	100%	5	4.0%		10	8.0%	110	88.0%
**Sex (n, %)**									
Male	59	47.2%	2	3.4%		8	13.6%	49	83.1%
Female	66	52.8%	3	4.5%	0.552	2	3.0%	61	92.4%
**Age (years)**									
Mean, Median (years)	36.2, 34.1		31.0, 26.1			37.5, 36.9		36.3, 34.0	
Range (years)	18 – 79.8		18.1 – 50.2			23.7 – 57.6		18 – 79.8	
**Other Symptoms (n, %)**									
Additional DD/ID/D	78	62.4%	3	3.8%		6	7.7%	69	88.5%
No DD/ID/D	47	37.6%	2	4.3%	0.623	4	8.5%	41	87.2%
**Severity of Obesity (n, %)**									
Milder Obesity	40	32.0%	3	7.5%	0.308	6	15.0%	31	77.5%
Severe Obesity	40	32.0%	1	2.5%		2	5.0%	37	92.5%
BMI unknown	45	36.0%	1	2.2%		2	4.4%	42	93.3%

Supplementary Table 4 - PanelApps

ALL SOLVED CASES:

Number of Patients	Patients IDs	Gene / Locus	Full Gene name	Included in combined panel (PanelApp AUS + UK)?
8	8 – 15	*MC4R*	Melanocortin 4 receptor	yes
3	16 – 18	*PHIP*	Pleckstrin homology domain-interacting protein	yes
3	22 – 24	*SRRM2*	Serine/arginine repetitive matrix protein 2	no
3	9[a], 27, 28	16p11.2, proximal		no
2	29, 30	16p11.2, distal		yes
2	1, 2	*ADNP*	Activity-dependent neuroprotector homeobox	no
2	19, 20	*RAI1*	Retinoic acid-induced gene 1	no
1	3	*ALMS1*	Alstrom syndrome 1	yes
1	4	*CREBBP*	CREB-binding protein	no
1	5	*GNAS*	eotide-binding protein,alpha stimulating activity	yes
1	6	*LEPR*	Leptin receptor	yes
1	7	*MAGEL2*	MAGE-like 2	yes
1	21	*SPG11*	Spastic paraplegia 11	no
1	25	*TRIP12*	Thyroid hormone receptor interactor 12	no
1	26	2p25.3 (*MYT1L*)	(Myelin transcription factor 1-like)	yes
				**PATIENT SUM: 18/30 = 60.0%**

[a]: patient both with MC4R-variant and proximal 16p11.2 deletion

NON-SYNDROMIC:

Number of Patients	Patients IDs	Gene / Locus	Included in combined panel (PanelApp AUS + UK)?
6	8 – 10, 12, 13, 15	*MC4R*	yes
2	29, 30	16p11.2, distal	yes
1	9[a]	16p11.2, proximal	no
1	24	*SRRM2*	no
			**PATIENT SUM: 8/9 = 88.9%**

[a]: patient both with MC4R-variant and proximal 16p11.2 deletion

SYNDROMIC:

Number of Patients	Patients IDs	Gene / Locus	Included in combined panel (PanelApp AUS + UK)?
3	16 – 18	*PHIP*	yes
2	1, 2	*ADNP*	no
2	11, 14	*MC4R*	yes
2	19, 20	*RAI1*	no
2	22, 23	*SRRM2*	no
2	27, 28	16p11.2, proximal	no
1	3	*ALMS1*	yes
1	4	*CREBBP*	no
1	5	*GNAS*	yes
1	6	*LEPR*	yes
1	7	*MAGEL2*	yes
1	21	*SPG11*	no
1	25	*TRIP12*	no
1	26	2p25.3 (*MYT1L*)	yes
			**PATIENT SUM: 10/21 = 47.6%**
